# Advancements in research on the immune-inflammatory mechanisms mediated by NLRP3 inflammasome in ischemic stroke and the regulatory role of natural plant products

**DOI:** 10.3389/fphar.2024.1250918

**Published:** 2024-03-27

**Authors:** Kailin Yang, Liuting Zeng, Qi He, Shanshan Wang, Hao Xu, Jinwen Ge

**Affiliations:** ^1^ Key Laboratory of Hunan Province for Integrated Traditional Chinese and Western Medicine on Prevention and Treatment of Cardio-Cerebral Diseases, School of Integrated Chinese and Western Medicine, Hunan University of Chinese Medicine, Changsha, China; ^2^ Hunan Academy of Chinese Medicine, Changsha, Hunan, China; ^3^ Graduate School, Chinese Academy of Medical Sciences and Peking Union Medical College, Beijing, China; ^4^ Department of Critical Care Medicine, People’s Hospital of Ningxiang City, Ningxiang, China

**Keywords:** ischemic stroke, NLRP3 inflammasome, immune mechanism, inflammatory mechanism, natural plant products

## Abstract

Ischemic stroke (IS) is a major cause of mortality and disability among adults. Recanalization of blood vessels to facilitate timely reperfusion is the primary clinical approach; however, reperfusion itself may trigger cerebral ischemia-reperfusion injury. Emerging evidence strongly implicates the NLRP3 inflammasome as a potential therapeutic target, playing a key role in cerebral ischemia and reperfusion injury. The aberrant expression and function of NLRP3 inflammasome-mediated inflammation in cerebral ischemia have garnered considerable attention as a recent research focus. Accordingly, this review provides a comprehensive summary of the signaling pathways, pathological mechanisms, and intricate interactions involving NLRP3 inflammasomes in cerebral ischemia-reperfusion injury. Moreover, notable progress has been made in investigating the impact of natural plant products (e.g., Proanthocyanidins, methylliensinine, salidroside, α-asarone, acacia, curcumin, morin, ginsenoside Rd, paeoniflorin, breviscapine, sulforaphane, etc.) on regulating cerebral ischemia and reperfusion by modulating the NLRP3 inflammasome and mitigating the release of inflammatory cytokines. These findings aim to present novel insights that could contribute to the prevention and treatment of cerebral ischemia and reperfusion injury.

## 1 Introduction

Stroke, the second most lethal disease globally, is a highly disabling condition causing approximately 6 million deaths annually (Feske, 2021). In China, the number of stroke patients exceeds 13 million, a figure projected to surpass 30 million by 2030, imposing a substantial burden on patients and their families (Hussain et al., 2021; Saini et al., 2021). Stroke manifests as either ischemic stroke (IS), resulting from embolic blockage of cerebral artery blood flow, or hemorrhagic stroke caused by blood vessel rupture within the brain. Pathophysiological processes underlying stroke encompass disorders in bioenergy metabolism, cellular ion homeostasis imbalances, acidosis, elevation of intracellular calcium concentration, excitotoxicity, superoxide dismutase-mediated neurotoxicity, increased arachidonic acid expression, cytokine-mediated cytotoxicity, complement system activation, glial cell activation, leukocyte infiltration, and disruption of the blood-brain barrier. Inflammation constitutes a critical pathogenic mechanism in IS, as the inflammatory response can trigger both ischemic brain injury and facilitate tissue repair following damage (Qiu et al., 2021). Activated neurons, astrocytes, microglia, and endothelial cells in IS release pro-inflammatory factors such as tumor necrosis factor-α (TNF-α), interleukin-1β (IL-1β), IL-6, and IL-18, ultimately leading to neuronal and glial cell death (Ajoolabady et al., 2021; Zhu et al., 2022a). Recent studies have highlighted the potential importance of NOD-like receptors Pyrin Containing 1 (NLRP1) and NLRP3 inflammasome in neurons and glial cells for detecting IS-induced cell damage and immune response regulation (Xu et al., 2021; Wang et al., 2022; Zhang et al., 2022). The human NLRP1 inflammasome is composed of four cytoplasmic components, including NLRP1, an apoptosis-associated speck-like protein (ASC) harboring N-terminal pyrin domain (PYD) and caspase recruitment domain (CARD), a caspase-1 precursor, and either a caspase-4 precursor or a caspase-5 precursor (Piancone et al., 2021). The composition of the NLRP1 inflammasome in mice slightly differs and consists of NLRP1, ASC, caspase-1 precursor, caspase-11 precursor (homologous to caspase-4 precursor and caspase-5 precursor), and X-linked inhibitor of apoptosis (XIAP) (Huang et al., 2021a). On the other hand, the NLRP3 inflammasome comprises three cytoplasmic components, namely, NLRP3, ASC, and caspase-1 precursor (Sharma and Kanneganti, 2021). Although the mechanisms underlying NLRP1 and NLRP3 inflammasome activation in IS remain incompletely understood, several pathways potentially implicated in their stimulation have been identified, including ATP-mediated activation, acidosis-mediated activation, cathepsin-mediated activation, potassium-mediated activation, ROS-mediated activation, calcium ion-mediated activation, and cell edema-mediated activation (Pandey et al., 2021; Zhang et al., 2021a; Liu et al., 2021).

Following ischemic stroke (IS), the NLRP3 or NLRP1 protein forms a multi-protein complex known as the NLRP3 or NLRP1 inflammasome by binding with Caspase-1 precursor and ASC (Lin et al., 2021). Once formed, the inflammasome activates the caspase-1 precursor through inter-neighbor autocatalysis. Activated caspase-1 then cleaves the precursors of IL-1β and IL-18, generating active mature forms that are subsequently released into the extracellular environment to bind to corresponding cell membrane receptors. This triggers downstream MAPK and nuclear factor kappa B (NF-κB) pathways, leading to the re-transcription of IL-1β and IL-18 precursors, NLRP1, and NLRP3 (Li et al., 2022a). In IS, excessive production of IL-18 by neurons and glial cells induces an increase in IFN-γ levels and the release and maturation of IL-1β, promoting a state of brain tissue inflammation and causing severe damage (Mi et al., 2022). Additionally, activated caspase-1 can cleave and activate caspase-3 and caspase-7, leading to apoptosis through the internal and external pathways of cells, respectively (Wang et al., 2022). Recent studies have highlighted the regulatory role of natural plant products in modulating the cerebral ischemic inflammasome (Tao et al., 2022). This discovery opens up new avenues for exploring lead compounds as inflammasome inhibitors. This review aims to summarize the mechanisms of the inflammasome in IS and the regulatory effects of natural plant products. These findings serve as a theoretical reference for future drug development and clinical treatment.

## 2 Inflammation as the core pathological process of IS

Inflammation serves as the innate immune response of the body, playing a crucial role in eliminating harmful stimuli and promoting tissue repair initiation (Lee et al., 2018). The inflammatory response elicited by ischemic stroke (IS) extends throughout the entire IS process, starting from the activation of endothelial cells shortly after IS onset to the post-injury repair phase occurring several days to months later. It represents one of the key factors influencing neuronal death. However, an exaggerated inflammatory response can lead to significant harm. In the presence of cerebral ischemia, activated cells (including neurons, astrocytes, and endothelial cells) release pro-inflammatory cytokines such as IL-1β, IL-6, and IL-18, which induce the death of neurons and glial cells. Microglia in the central nervous system (CNS) are the first to be activated, secreting numerous inflammatory mediators and exacerbating the inflammatory response in the ischemic region, damaging the blood-brain barrier, and releasing various danger-associated molecular patterns (DAMPs) (Tang et al., 2023). These factors collectively induce the recruitment of peripheral immune cells to the lesion and its surroundings, extensively contributing to the inflammation and immune response in IS. Recent work by Dai et al. (2020) demonstrated that D-carvone attenuated the cerebral ischemia-reperfusion-induced inflammatory response in rats, reducing damage to the hippocampus and cortex. Additionally, released pro-inflammatory cytokines can induce the expression of adhesion molecules such as intercellular adhesion molecule-1 (ICAM-1), vascular cell adhesion molecule (VCAM), selectins (e.g., P-selectin, E-selectin), and integrins. These adhesion molecules are crucial for the infiltration of immune cells, especially neutrophils and monocytes/macrophages, into the ischemic area during reperfusion. Unfortunately, this often leads to secondary injuries in ischemia-reperfusion injury. Moreover, activated neurons and glial cells release monocyte chemotactic protein-1 (MCP-1/CCL2), promoting leukocyte migration to damaged tissues. However, a recent study revealed that the accumulation of leukocytes in nerves and blood vessels did not spatially correlate with increased vascular permeability or enhanced expression of endothelial cell adhesion molecules (Enzmann et al., 2013). Although the mechanism underlying ischemia-reperfusion injury remains unclear, it has been observed that leukocyte infiltration can lead to the release of various cytotoxic agents, including additional pro-inflammatory cytokines (e.g., IL-1β, IL-6), reactive oxygen species (ROS) produced by reduced nicotinamide adenine dinucleotide phosphate oxidase, as well as nitric oxide, induced by inducible nitric oxide synthase (iNOS) and matrix metalloproteinases (MMP). These MMPs can contribute to extracellular matrix damage and blood-brain barrier disruption, ultimately exacerbating cerebral edema and hemorrhage, leading to neuronal and glial cell death (Shen et al., 2023).

## 3 Inflammasome participates in the pathological process of IS

**FIGURE 1:**
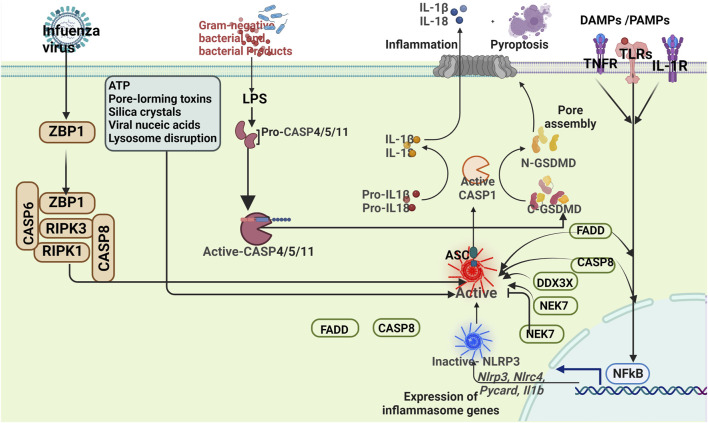
Mechanisms of the inflammasome.

The inflammasome is characterized by three components: a receptor protein, ASC, and Pro-caspase-1. Among the pattern recognition receptors investigated, the most extensively studied ones are nucleotide-binding and oligomerization domain-like receptors (NLRs) and absent in melanoma 2-like receptors (ALRs) (Huang et al., 2021a; Pandey et al., 2021). The NLR family comprises five subtypes: NLRA with an acidic transactivation domain, NLRB with baculovirus inhibitor repeat, NLRC with CARD (NOD1, NOD2, NLRC3-5), NLRPs containing protein domains (NLRP1-14), and NLRXs with unknown domains (Li et al., 2021a; Zhang et al., 2021b). The ALR family consists of AIM2 protein and IFN-γ inducible protein 16 (IFI16) protein (Zheng et al., 2021). Extensively studied inflammasomes include NLRP1, NLRP3, NLRC4, and AIM2 (Zheng et al., 2021). Several studies have reported the significant involvement of NLR containing pyrin domain (NLRP) in the inflammatory response among ischemic stroke patients (Seoane et al., 2020). The mechanism of the inflammasome was summarized in Figure 1.

### 3.1 NLRP1

The NLRP1 inflammasome consists of the NLRP1 receptor, ASC and Pro-caspase-1, is a member of the NLRs family, and is highly expressed in brain tissue. The NLRP1 receptor is characterized by 5 domains: PYD, nucleotide-binding oligomerization (NACHT), C-terminus is leucine repeat domains (LRRs), function-to-find domain (FIIND) and CARD. When the NLRP1 receptor is activated, the FIND domain is automatically cleaved (Ciążyńska et al., 2021; de Brito Toscano et al., 2021). Cao et al. showed that NLRP1 is a target gene of miR-9a-5p and is involved in the NLRP1 inflammasome-mediated inflammatory response induced by ischemic injury (Cao et al., 2020). Overexpression of miR-9a-5p downregulates NLRP1. Overexpression of miR-9a5p not only reduced the levels of NLRP1, Asc and Pro-caspase-1, but also decreased the levels of IL-1β and IL-18, suggesting that overexpression of miR-9a5p can improve brain injury after IS (Cao et al., 2020).

### 3.2 NLRP3

The NLRP3 inflammasome consists of the NLRP3 receptor, ASC, and Pro-caspase-1. The NLRP3 receptor, a member of the NLR family, exhibits three structural features: N-terminal PYD, central NACHT, and C-terminal LRR domains (Zhang et al., 2021a; Zhao et al., 2021). The N-terminal PYD domain enables interaction with the downstream adaptor protein ASC via dual ligand binding. The NACHT domain regulates the activation of NLRP1 and NLRP3 receptors, initiating oligomerization and formation of the central core of the inflammasome, an ATP-dependent process (Levinsohn et al., 2012). The LRR domain is believed to participate in ligand sensing and autoregulation (Levinsohn et al., 2012). Recent studies have explored potential interventions targeting the NLRP3 inflammasome for neuroprotection in ischemic stroke. For instance, Ma et al. discovered that salvianolic acid for injection can inhibit the activation of microglial NLRP3 inflammasome, facilitate the transition of microglial phenotype from M1 to M2, and reduce neuronal apoptosis, thereby exerting neuroprotective effects (Ma et al., 2021). Yin et al. also revealed the involvement of TET2 in the inflammatory response induced by cerebral ischemia-reperfusion through the demethylation of TUG1 and regulation of the TUG1/miR-200A-3p/NLRP3 pathway (Yin et al., 2021). Sun et al. identified the role of the low-density lipoprotein receptor (LDLR) in mediating NLRP3-dependent neuronal pyroptosis and neuroinflammation after ischemic stroke, suggesting LDLR as a potential therapeutic target for neuroinflammatory responses in acute cerebral ischemic injury (Sun et al., 2020). Assembly and activation of the NLRP3 inflammasome rely on two complementary signals associated with cellular damage. One initiation signal involves the NF-κB and mitogen-activated protein kinase (MAPK) signaling pathways. These pathways upregulate the expression of NLRP3 inflammasome complex proteins, precursor IL-1β, and precursor IL-18 (Paerewijck and Lamkanfi, 2022). The other complementary signal mediates NLRP3 activation and ASC phosphorylation, initiating NLRP3 inflammasome assembly, caspase-1 activation, and processing of Pro-IL-1β and Pro-IL-18, ultimately resulting in the secretion of IL-1β and IL-18 (Takahashi, 2022).

#### 3.2.1 Initiation regulation of NLRP3 inflammasome activation

In the resting state, the expression levels of NLRP3, Pro-IL-1β and Pro-IL-18 in cells are low, and they cannot directly assemble or activate the NLRP3 inflammasome. Therefore, upregulation of priming signals, especially NLRP3-related proteins, is required for activation of the NLRP3 inflammasome (Coll et al., 2022). Fann et al. found that intravenous injection of immunoglobulin preparations could reduce the activation of NF-κB and MAPK signaling pathways, resulting in decreased expression and activation of NLRP3 inflammasomes (Fann et al., 2018). This suggests that NF-κB and MAPK signaling pathways play critical roles in regulating the expression and activation of the NLRP3 inflammasome in primary cortical neurons and brain tissue under ischemic conditions (Huang et al., 2021a).

#### 3.2.2 Activation regulation of NLRP3 inflammasome activation

After activation of the priming signal, the adaptor protein ASC recruits the NLRP3 protein to form the NLRP3 inflammasome complex (Chen et al., 2021a). Currently, the NLRP3 inflammasome has multiple activation mechanisms, including K+ efflux, ROS overproduction, mitochondrial dysfunction, Ca overload, and lysosomal rupture (Milner et al., 2021). Among the extracellular and intracellular factors, K+ efflux plays an important role in activating the NLRP3 inflammasome (Muñoz-Planillo et al., 2013). This non-selective K+ cation channel located on the cell surface can change intracellular ion content depending on the binding of adenosine triphosphate, activate downstream signals, and induce the maturation and secretion of IL-1β. Studies have shown that downregulation of intracellular K+ levels is essential for activation of the NLRP3 inflammasome pathway compared to other known stimuli, highlighting the important role of K+ efflux in this process. Purinergic ion channel receptor 7 (P2X7R) is one of the receptors associated with K+ efflux, and its activation may induce the release of proinflammatory cytokines and amplify ischemic injury via ATP (Kelley et al., 2019). Ye et al. found that the expressions of P2X7R, NLRP3 inflammatory components and cleaved caspase-3 were significantly enhanced in ischemic brain tissue after stroke (Ye et al., 2017). However, the expression of cleaved caspase-3 was significantly attenuated after stroke treatment with a P2X7R antagonist (BBG) or an NLRP3 inhibitor (MCC950), which significantly reduced brain infarct volume, neuronal apoptosis, and nerve damage. Mitochondrial damage is another important activation mechanism of the NLRP3 inflammasome. Mitochondria are double-membrane-bound organelles that are the main site for energy and ROS production in cells (Peng and Jou, 2010). Previous studies have shown that under various cellular stresses, especially high levels of ROS produced by mitochondria activate the NLRP3 inflammasome signaling pathway (Heid et al., 2013). A large amount of ROS-induced ROS scavenger thioredoxin is cleaved from thioredoxin-interacting protein (TXNIP), which then directly binds to NLRP3 protein and regulates its assembly through oligomerization (Yin et al., 2017). Ishrat et al.showed that the expression of TXNIP in brain tissue increased after IS, and ROS can lead to the dissociation of TXNIP and thioredoxin 1 (TRX1), which can quickly bind to NLRP3, induce the activation of NLRP3 inflammasome, inhibit the activation of NLRP3 inflammasome by inhibiting TXNIP, and reduce ischemic brain injury (Ishrat et al., 2015). Dysfunctional mitochondria also release mitochondrial DNA into the cytoplasm, which directly induces the assembly of the NLRP3 inflammasome complex through molecular self-association (Ip and Medzhitov, 2015).

### 3.3 AIM2

The AIM2 inflammasome is a cytoplasmic sensor that recognizes double-stranded DNA (dsDNA) from viruses, bacteria, or the host itself. AIM2 consists of PYD and HIN-200 domains, and AIM2 protein is the activating component of the AIM2 inflammasome. Li et al. found that cytosolic DNA participates in a variety of independent but complementary DNA sensing signals through cyclic adenosine monophosphate synthase and AIM2, and synergistically exerts the greatest inflammatory response during ischemia (Li Q. et al., 2020a). The inhibitor of cytoplasmic double-stranded DNA, A151, significantly reduced cerebral infarct volume, alleviated neurological deficits, and reduced cell death, inhibited the overall neuroinflammatory response, and may provide a new therapeutic concept for IS. Liang et al. found that MEG3 knockout could inhibit oxygen-glucose deprivation/reoxygenation-induced pyroptosis and inflammatory responses (Liang et al., 2020). Lack of MEG3 inhibits caspase-1 signaling and reduces the expression of AIM2, ASC, cleaved caspase-1 and GSDMD-N. This suggests that the MEG3/miR-485/AIM2 axis is involved in pyroptosis by activating the caspase-1 signaling pathway during cerebral ischemia-reperfusion, and may be an effective therapeutic target for IS (Liang et al., 2020).

### 3.4 NLRC4

NLRC4, also known as IL-1β converting enzyme protease activating factor (IPAF), is a member of the NLR family (Sundaram and Kanneganti, 2021). The NLRC4 receptor is characterized by three domains: its N-terminus is the caspase recruitment domain (CARD), which is an effector domain that recruits and activates Pro-caspase-1 and is responsible for downstream signal transduction. In the middle is the NACHT domain, which is a characteristic domain shared by members of the NLR family, NBD-HD1-WHD-HD2 from the N-terminal to the C-terminal, which can mediate the oligomerization of NLR molecules and change its configuration. The LRRs, which are responsible for the recognition and binding of ligands such as PAMP (Andrade and Zamboni, 2020; Bauer and Rauch, 2020). Recent studies have shown that inflammasome activation can lead to the death of neurons and glial cells, which in turn leads to brain damage and nervous system damage after ischemic stroke (Wen et al., 2021a). Wang et al. found that in microglia induced by high glucose hypoxia/reoxygenation (H/R), knockout of the long non-coding RNA-Fendrr (LncRNA-Fendrr) gene reduced NLRC4 and inflammatory cytokines (Wang et al., 2021a). LncRNA-Fendrr can protect the ubiquitination and degradation of NLRC4 protein through the E3 ubiquitin ligase HERC2, thereby accelerating the pyroptosis of microglia.

**FIGURE 2:**
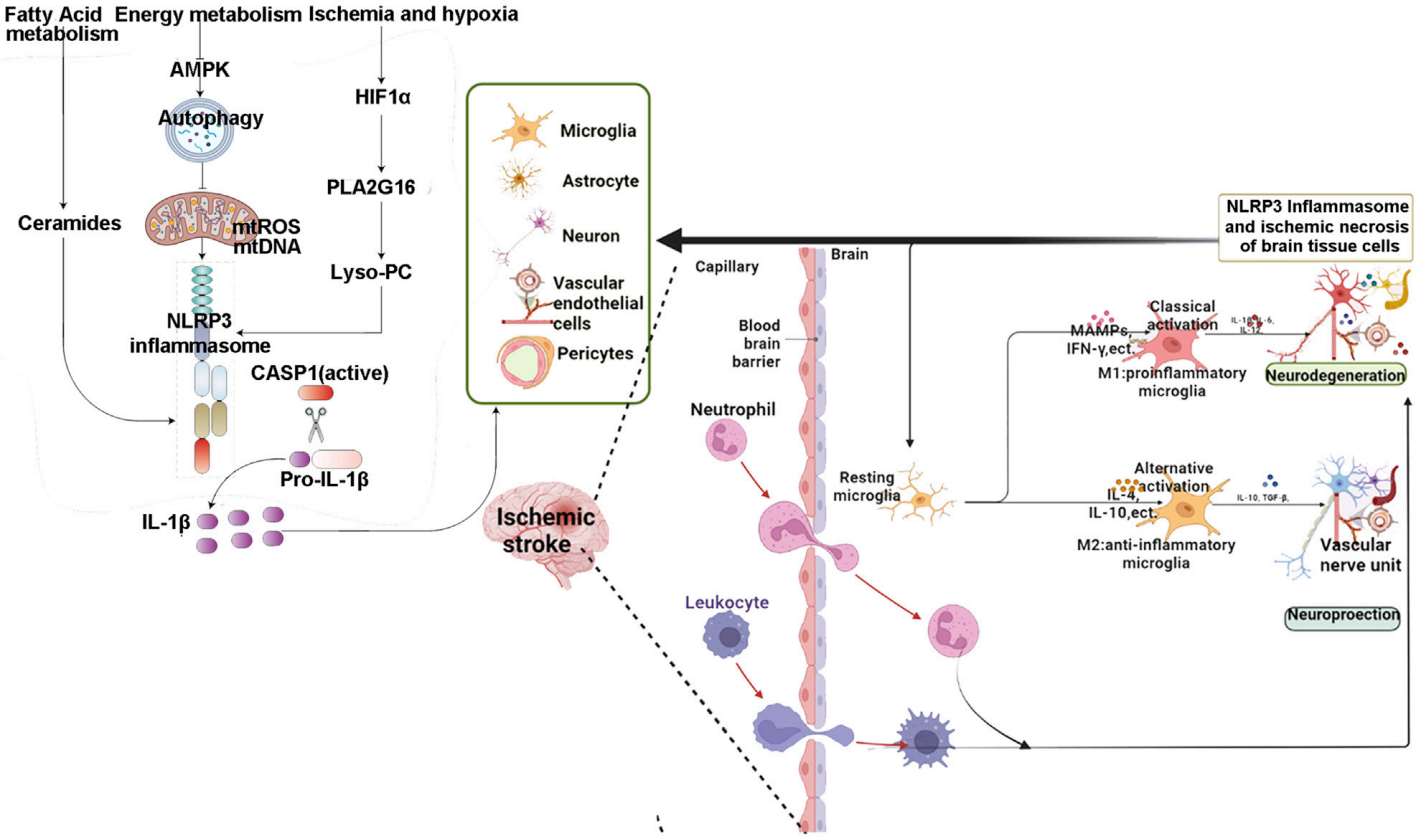
The mechanisms of inflammasome involvement in IS.

The mechanisms of inflammasome involvement in IS were summarized in Figure 2.

## 4 Inflammasomes involves in cerebral ischemia-induced cell damage

### 4.1 Inflammasome and apoptosis

Apoptosis is an active process involving activation, expression and regulation of a series of genes. Activation of classical apoptosis occurs mainly through two pathways (Christgen et al., 2022). One is the extrinsic pathway, which is activated by activating apoptosis receptors on the cell surface, ultimately activating caspase-8 or caspase-10 (Gudipaty et al., 2018). The other is the endogenous pathway, also known as the mitochondrial apoptosis pathway, which starts with mitochondrial cytochrome C and activates caspase-9 (Tang et al., 2019a). Both pathways lead to a signal transduction cascade that ultimately leads to apoptosis through activation of caspase-3 (Wu et al., 2020a). Previous studies have shown that free radicals generated by cerebral ischemia-reperfusion are mainly released by mitochondria, leading to oxidative stress in neurons. Excessive production of reactive oxygen species in mitochondria can damage mitochondria and lipids, thereby impairing mitochondrial function and leading to increased permeability. Increased permeability of mitochondria releases cytochrome C, which activates caspases and leads to cell death after cerebral ischemia-reperfusion (DArcy, 2019). Kang et al. found that TRIM22 gene silencing inhibited the activation of NF-κB/NLRP3, thereby inhibiting neuroinflammation and apoptosis, indicating that TRIM22 may be a potential target for the treatment of brain I/R injury (Kang et al., 2021).

### 4.2 Inflammasome and autophagy

In the process of autophagy, autophagic vesicles are formed in cells, which wrap their own proteins or organelles, and then fuse with lysosomes and degrade them. There is a close relationship between the NLRP3 inflammasome and autophagy (Nakatogawa, 2020). On the one hand, the activation of NLRP3 inflammasome can regulate the induction of autophagy (Zhao and Zhang, 2019); on the other hand, autophagy can control the activation of inflammasome and its activity (Deretic et al., 2013). Together, they regulate the balance between host defense, inflammatory responses, and prevention of excessive inflammation, and form the necessary positive and negative feedback loops. Activation of caspase-1 inhibits autophagy induction and activates the inflammatory response, which is required for pathogen clearance (Kimmey and Stallings, 2016). However, excessive inflammatory responses can lead to organ and tissue damage and induce inflammatory diseases. Autophagy suppresses inflammatory responses by removing NLRP3 inflammasome activators and inflammatory components (Chang et al., 2021). Wang et al. found that inhibition of GSK-3β could downregulate NLRP3 expression by enhancing autophagy activity in cerebral ischemia-reperfusion injury, suggesting that GSK-3β may be its specific target (Wang et al., 2019). He et al. found that cerebral ischemia-reperfusion injury activates the NLRP3 inflammasome, increases the levels of caspase-1, IL-1β and IL-18, and enhances autophagy activity. Resveratrol is a specific NAD-dependent deacetylase sirtuin-1 (Sirt1) agonist. Cerebral infarct volume and cerebral water content decreased and neurological scores improved after resveratrol treatment. Intraventricular injection of 3-MA to inhibit autophagy blocked the inhibitory effect of resveratrol on NLRP3 inflammasome activation. Knockdown of Sirt1 significantly blocked resveratrol-induced autophagy activity and inhibition of NLRP3 inflammasome activation, and upregulated autophagy. This suggests that resveratrol has a protective effect on cerebral ischemia-reperfusion injury by inhibiting the activation of NLRP3 inflammasome by inhibiting Sirt1-dependent autophagy activity (He et al., 2017).

### 4.3 Inflammasome and pyroptosis

Pyroptosis is a programmed cell death that occurs in two main ways. The classical pyroptotic pathway of caspase-1 is that the pyrin domain of the NLR family binds to ASC by recognizing cognate ligands, and then binds and activates the precursor of caspase-1 to form active caspase-1 (Rao et al., 2022). Activated caspase-1 cleaves GSDMD into a 22 kDa C-terminal fragment and a 31 kDa N-terminal fragment. The production of GSDMD-N directly induces cell membrane perforation and rupture, and the release of cellular contents triggers an inflammatory response (Feng et al., 2022). Meanwhile, activated IL-1β and IL-18 proteins are released to the outside of the cell through stomata on the cell membrane (Xu et al., 2017). Under the stimulation of various infectious factors represented by Gram-negative surface endotoxin (LPS), the non-classical pathway of pyroptosis will be activated. Caspase-11 has been shown to bind to LPS as a natural receptor, and LPS can enter the cell in the form of endocytosis and bind to and activate the caspase-11 precursor. Active IL-1β and IL-18 are released along with the rupture and perforation of the cell membrane, triggering an inflammatory response. Meanwhile, the N-terminal fragment of GSDMD activates NLRP3 through the canonical pathway, thereby inducing pyroptosis (Chen et al., 2018). Li et al. found that indobufen or aspirin pretreatment combined with clopidogrel or ticagrelor can reduce rat cerebral ischemia-reperfusion and PC12 cell oxygen glucose deprivation/reoxygenation inflammatory corpuscle mediated pyroptosis by inhibiting the NF-κB/NLRP3 signaling pathway (Li F. et al., 2021b). Fann et al. (2018) showed that when cerebral ischemia-reperfusion injury occurs, peripheral inflammatory cells can release pro-inflammatory cytokines such as IL-18 and IL-1β through the blood-brain barrier and activated microglia in the central system. This resulted in high expression of pyroptosis-related proteins, resulting in extensive glial and neuronal cell death, suggesting that pyroptosis plays an important role in the process of cerebral ischemia-reperfusion injury. Inhibition of NLRP3 inflammasome expression can simultaneously inhibit the expression of downstream pyroptosis pathway-related proteins, limit the inflammatory response and alleviate cerebral ischemia-reperfusion injury. Therefore, therapeutic interventions targeting neuronal inflammasome activation may provide new opportunities for the treatment of IS.

## 5 Modulation of inflammasome in IS by natural plant products

### 5.1 Natural compounds

#### 5.1.1 Toona polyphenols

The fruit of Toona sinensis is a member of the Meliaceae and is commonly used to prepare tea to prevent various diseases. Its main active ingredients are polyphenolic compounds. In recent years, numerous studies have confirmed the beneficial effects of Toona sinensis in treating various diseases, including central nervous system diseases, cardiovascular diseases, and diabetes (Zhao et al., 2023a; Wu et al., 2023). Zhao et al. found that Toona polyphenols can reduce the infarct volume and improve neural function in mice with chronic intermittent hypoxia (CIR). The optimal protection dosage is 100 mg/kg. The mechanism of protection may involve regulation of the prefrontal cortex and hippocampal MAPK pathways, NLRP3 inflammasome pathways, and inhibition of neural inflammation and cell apoptosis (Zhao, 2022).

#### 5.1.2 Proantho cyanidins (PC)

PC are a class of large flavonoid polyphenols widely found in the plant kingdom (Gupta et al., 2022). Their molecular structure contains multiple hydroxyl structures that can effectively combine with oxygen radical anions to exhibit antioxidant and reduce inflammation properties. Since the 1980s, multiple studies have shown that PC extracts from various plants have pharmaceutical and biological activities, including reducing inflammatory responses, treating antioxidant therapy, antineoplastic therapy, and protecting the cardiovascular system (Pinent et al., 2006; Holt et al., 2012; Lee, 2017; Osakabe et al., 2022). Yang et al. found that PC can inhibit the activation of the TLR4-NLRP3 signaling pathway and the upregulation of caspase-1 and IL-1β in BV2 cells induced by OGD/R. The results of the *in vitro* experiment were consistent with those obtained using Cli-095 pretreatment. The study concluded that PC can protect neurons *in vitro* by downregulating the TLR4-NLRP3 inflammatory signaling pathway induced by OGD/R, and the protection is related to time and concentration (Yang, 2022).

#### 5.1.3 Panax notoginseng saponins (PNS)

PNS is the main active ingredient in Sanqi, with the functions of dilating arteries, inhibiting platelet aggregation, reducing blood lipid levels, relieving inflammation, and antioxidant activity (Xie et al., 2018; Liu et al., 2020). Pharmacological studies have shown that PNS can reduce the release of chemotactic cytokines such as tumor necrosis factor-α (TNF-α) and interleukin-1 (IL-1), inhibit the activation of nuclear factor-κB (NF-κB), and the formation of NLRP3 inflammasomes, thereby suppressing inflammation responses (Song et al., 2017; Xu et al., 2018; Qu et al., 2020). PNS also protects myocardial mitochondria and reduces the leakage of lactate dehydrogenase (LDH) to improve myocardial energy metabolism. PNS can also inhibit the abnormal activation of platelets and the abnormal activation of the coagulation system to improve microcirculatory flow. PNS increases the activity of endogenous antioxidant enzymes and reduces the production of active oxygen to alleviate oxidative stress responses. PNS also regulates the concentration of intracellular calcium ions and reduces the abnormal expression of cancer genes to inhibit cell apoptosis. Zhou et al. found that the mechanism by which PNS improves I/RI may be related to the downregulation of NF-κB protein activation, the reduction in NLRP3 inflammasome formation, and the downregulation of chemotactic cytokines such as IL-1β secretion (Zhou et al., 2021).

#### 5.1.4 Neferine

Neferine is a dimeric quinolinone alkaloid extracted from the embryo of the mature seeds of the water chestnut plant *Nelumbo nucifera* (MarthandamAsokan et al., 2018), a member of the Nelumbonaceae family. Neferine has various effects such as anti-atherosclerosis, anti-hypertension, anti-thrombosis, anti-diabetic vascular complications, and vascular protection, as well as anti-arrhythmia (Tang et al., 2019b; Bharathi Priya et al., 2021; Qi et al., 2021). Studies have shown that Neferine has good therapeutic effects and low side effects in the treatment and prevention of cardiovascular and cerebrovascular diseases. Huang et al. found that methylnelumbellate can alleviate brain ischemic reperfusion injury and immune dysregulation in mice by inhibiting the activation of NLRP3 inflammasome (Huang et al., 2021).

#### 5.1.5 Salvianolic acid

Salvianolic acid has the function of blood purification and is used for cerebral infarction (Xiao et al., 2020). The main chemical components of salvianolic acid include D-(−)-pinoresinol-β-D-glucoside, rosmarinic acid, p-coumaric acid, ferulic acid, pinoresinol, and salvianolic acid B water-soluble phenolic acids (Guo and Wang, 2022). Modern pharmacological studies have shown that salvianolic acid has analgesic, anti-inflammatory, antioxidant, neurotrophic, and neuroprotective effects (Wu et al., 2020b; Xin et al., 2020), which can significantly improve the nerve function damage in patients with ischemic stroke, restore their daily life behavior, and improve the prognosis and quality of life (Lyu et al., 2019). Ma et al. found that salvianolic acid may inhibit the activation and cell apoptosis of small glial cells through the P2X7/NLRP3/GSDMD pathway, thus reducing the downstream inflammatory cascade reaction and exerting a nerve protection effect on experimental brain ischemic reperfusion injury (Ma, 2021).

#### 5.1.6 Salidroside

Salidroside, the main active ingredient in Rhodiola rosea, has been shown to possess broad biological activities, including anti-inflammatory, antioxidant, antitumor, immune regulatory, and protective effects on the lungs, kidneys, liver, cardiovascular system, and nervous system (Xu et al., 2019; Qu et al., 2022). In terms of cerebral ischemia, Salidroside can improve energy metabolism disorders, metabolic acidosis, Ca2+ overload, and inhibit oxidative stress, inflammatory response, cell apoptosis, and autophagy (Fan et al., 2020; Magani et al., 2020; Zhang et al., 2021d). Liu et al. found that Salidroside may inhibit the activation of NLRP3 inflammasome and cell apoptosis in microglia by suppressing the TLR4/NF-κB signaling pathway, thereby reducing neuronal damage. Salidroside may also inhibit the activation of NLRP3 inflammasome and TLR4/NF-κB signaling pathway in rat cerebral ischemia-reperfusion injury by increasing the expression of miR-370-3p (Liu, 2022).

#### 5.1.7 α-Asarone

α-Asarone is the main active component of Acorus tatarinowii, a perennial herb of the Araceae family. Studies have found that besides having antitumor, insecticidal, antibacterial, antitussive, bronchodilatory, neuroprotective, antiepileptic, and antidepressant effects, α-Asarone and beta-asarone also exhibit good pharmacological activities in cardiovascular and cerebrovascular diseases, such as protecting myocardial and vascular cells (including endothelial cells and vascular smooth muscle cells), preventing thrombosis, lowering blood lipids, and improving vascular function (Chellian et al., 2017; Uebel et al., 2021; Uebel et al., 2021). In the context of cerebral ischemia, α-Asarone exerts its effects by alleviating energy metabolism and ion metabolism disorders, reducing excitotoxicity, oxidative stress, inflammatory factor expression, protecting the blood-brain barrier, and other mechanisms (Liu et al., 2019; Balakrishnan et al., 2022). Xu et al. found that α-Asarone has a significant protective effect against cerebral ischemia/reperfusion injury, mainly by regulating ROS activity, inhibiting NF-κB phosphorylation, reducing the excessive activation of NLRP3 inflammasomes, and exerting anti-inflammatory effects to protect against cerebral ischemia/reperfusion injury (Fei-Fei et al., 2022).

#### 5.1.8 Acacia

Acacetin is a natural flavonoid drug that can be extracted from various plants and has attracted the attention of many researchers due to its wide range of pharmacological effects (Wu et al., 2022). Increasing evidence indicates that acacetin has potential in the treatment of various diseases such as anti-tumor, cardioprotective, anti-inflammatory, and neuroprotective effects (Singh et al., 2020; Cui et al., 2022). In the case of cerebral ischemia, acacetin may reduce the permeability of the blood-brain barrier by increasing the expression of Occludin, Claudin-5, and ZO-1 proteins, thereby exerting a neuroprotective effect in cerebral ischemia-reperfusion injury (Niu and Yang, 2020). Bu et al. found that acacetin can increase the survival rate of astrocytes after OGD/R injury, reduce the release of LDH, decrease ROS generation, increase the expression of LC3-II and beclin-1 proteins, and further downregulate the expression of NLRP3, caspase-1, and IL-1β. In summary, Acacetin can alleviate OGD/R-induced damage to astrocytes, thereby exerting a protective effect on cerebral ischemia-reperfusion injury. Its mechanism of action may be related to the inhibition of ROS production, activation of autophagy, and inhibition of NLRP3 inflammasome (Juan et al., 2023).

#### 5.1.9 Curcumin

Curcumin is a polyphenolic substance extracted from the rhizomes of turmeric, which is commonly used as a food coloring agent. Curcumin has been found to possess a wide range of pharmacological activities, such as antibacterial, antioxidant, anti-apoptotic, anti-tumor, anti-oxidative stress, anti-inflammatory, anti-viral, lipid-lowering, liver-protective, bile-stimulating, and neuroprotective effects (Termini et al., 2020; Jabczyk et al., 2021). Curcumin has also been shown to improve age-related diseases such as atherosclerosis, diabetes, cardiovascular disease, and chronic kidney disease (Peng et al., 2021). Curcumin can exert neuroprotective effects in cerebral ischemia, intracerebral hemorrhage (ICH), and subarachnoid hemorrhage (SAH) through various pathways such as anti-inflammatory reactions, anti-oxidative stress, anti-apoptosis, and autophagy (Li et al., 2020b; Fan and Lei, 2022; Marques et al., 2022). Zhang et al. (2023) found that curcumin can reduce the volume of cerebral infarction and improve neurological function in rats, possibly by reducing the inflammatory response in the brain after cerebral infarction; the anti-inflammatory mechanism of curcumin may be related to its blockade of the NLRP3/Caspase-1 signaling axis, inhibition of NLRP3 inflammasome function, and reduction of the synthesis and release of inflammatory cytokines. Zhao et al., 2023b found that curcumin can alleviate cortical neuronal inflammation and synaptic injury caused by cerebral ischemia-reperfusion in rats by inhibiting inflammatory cytokine levels and reducing brain damage.

#### 5.1.10 Morin

Morin, a natural active substance extracted from various plants, belongs to the flavonoid class of compounds and is a secondary metabolite of phenols in plants, widely distributed in nature (Caselli et al., 2016). Morin displays its pharmacological activity by modulating various cell signaling pathways, such as nuclear factor-kappa B (NF-κB), mitogen-activated protein kinase (MAPK), Janus kinase/signal transducer and activator of transcription (JAKs/STATs), Kelch-like ECH-associated protein1/Nuclear erythroid-2-related factor (Keap1/Nrf2), endoplasmic reticulum (ER), mitochondria-mediated apoptosis, Wnt/β-catenin, and Rapamycin (mTOR) signaling pathways (Sinha et al., 2016; Rajput et al., 2021). Extensive evidence has shown that morin plays a beneficial role in various chronic and life-threatening degenerative diseases (Kataria et al., 2018; Solairaja et al., 2021). Xue et al. found that morin can alleviate cerebral ischemia-reperfusion injury in rats, inhibit neuronal apoptosis, and its mechanism may be related to the inhibition of TXNIP/NLRP3/Caspase-1 signaling pathway activation (Xue et al., 2023).

#### 5.1.11 Ginsenoside Rd

Ginsenoside Rd is one of the major active monomers of ginsenosides and belongs to the dammarane-type ginsenosides with a diol structure (Chen et al., 2022a). Although ginsenoside Rd is present in low concentrations in Panax species, it has attracted wide attention from scholars due to its strong biological activity. Studies have shown that ginsenoside Rd exhibits various pharmacological effects, such as protecting cardiovascular and renal functions, exerting anti-tumor and immunomodulatory activities, and showing good neuroprotective effects on the central nervous system (Li et al., 2022b; Tang et al., 2022). In terms of cerebral ischemia, ginsenoside Rd can improve ischemic stroke-induced damage by inhibiting oxidative stress and inflammation. Its mechanism mainly involves upregulating the endogenous antioxidant system, the phosphatidylinositol 3-kinase/protein kinase B and extracellular signal-regulated kinase 1/2 pathways, protecting mitochondrial membrane potential to prolong the survival of neuronal cells, inhibiting the activation of nuclear factor kappa B, transient receptor potential melastatin, acid-sensing ion channel 1a, poly(ADP-ribose) polymerase-1, and protein tyrosine kinase, as well as reducing the release of cytochrome c and apoptosis-inducing factor (Ye et al., 2013; Nabavi et al., 2015; Song et al., 2022). Yao et al. found that ginsenoside Rd can inhibit the expression levels of cell death-associated proteins, IL-18 and IL-1β secretion, intracellular ROS content, cell death ratio, and the interaction between TXNIP and NLRP3. Furthermore, ginsenoside Rd exerts a protective effect on neurons after cerebral ischemia/reperfusion injury, which is associated with upregulating the expression level of miR-139-5p, decreasing the expression levels of FoxO1 and Keap1, and activating the Nrf2 antioxidant signaling pathway, thereby inhibiting cell death induced by the ROS-TXNIP-NLRP3 inflammasome axis (Yao, 2022).

#### 5.1.12 Paeoniflorin

Paeoniflorin is the main active monomer component of Paeonia. Previous studies have shown that paeoniflorin exhibits various activities, such as anti-free radical damage, inhibition of intracellular calcium overload, and neuroprotection. *In vivo* experiments have demonstrated that paeoniflorin has effects on reducing blood viscosity, anti-platelet aggregation, vasodilation, improving microcirculation, and exerting antioxidative and anticonvulsant effects (Zhang and Wei, 2020; Zhou et al., 2020; Wang et al., 2021b). In cerebral ischemia research, paeoniflorin has been found to protect PC12 cells against calcium overload-induced injury. Additionally, paeoniflorin has been shown to protect against neurotoxicity induced by calcium overload in PC12 cells (Hu et al., 2019; Long et al., 2020; Tang et al., 2021). In terms of inhibiting the inflammasome, He et al. found that paeoniflorin has a protective effect on nerve damage in OGD-induced rat hippocampal slices, which may be related to the downregulation of the expression of NLRP3 and NLRP1 inflammasome components, thereby affecting the release of downstream inflammatory factors and cell apoptosis (He, 2016). A recent study also detected paeoniflorin in the colon and found that it improved UC symptoms in mice, inhibited the infiltration of macrophages in the mesentery and colon tissue, suppressed NLRP3 protein in colon macrophages, and inhibited the release of cytokine IL-1β (Liu et al., 2018). Moreover, a recent study showed that paeoniflorin can improve neuronal functional impairment mediated by ACI by inhibiting the activation of NLRP3 and Caspase-1, reducing microglial cell activation, and inhibiting neuronal necrosis (Aikmu et al., 2021).

#### 5.1.13 Astragaloside IV

Astragalus membranaceus (AM) is a commonly used Chinese herbal medicine for the treatment of cardiovascular and cerebrovascular diseases. It has the effects of promoting diuresis, reducing edema, tonifying qi, and enhancing the immune system (Zhang et al., 2020). Modern medicine has also shown that AM has the ability to reduce oxidative stress, inhibit apoptosis, and decrease edema (Zang et al., 2020; Chen et al., 2021b). Astragaloside IV (AST-IV) is the main active component of AM responsible for its cardiovascular effects and is used as an important indicator for the evaluation of AM content. Experimental studies have demonstrated that AST-IV can improve neurological function, reduce cerebral infarct volume, and decrease blood-brain barrier permeability, thus exerting a protective effect in cerebral ischemia. The mechanism is mainly related to its anti-oxidant, anti-inflammatory, and anti-apoptotic properties, which are achieved by inhibiting the expression of MPO, TNF- α, IL-1𝛽, iNOS, intracellular adhesion molecules, and NF-𝜅B (Tang et al., 2019c; Tang, 2019). These findings suggest that PNS and AST-IV can play a protective role in ischemic brain injury through multiple targets and pathways. Tang et al. used an improved thread embolization method to prepare a rat model of middle cerebral artery occlusion/reperfusion to evaluate the effect of AST-IV on cerebral ischemia-reperfusion injury. Compared with the model group, AST-IV significantly reduced the neurological function deficit score, cerebral infarct volume, and the protein levels of NLRP3, Caspase-1, pro-IL-1β, IL-1β, pro-IL-18, and IL-18 in brain tissue, and inhibited the expression of phosphorylated NF-κB protein. These results suggest that AST-IV has a protective effect against cerebral ischemia-reperfusion injury, and its mechanism may be related to the inhibition of NF-κB protein phosphorylation and the inhibition of NLRP3 inflammasome activation (Tang et al., 2019c; Tang, 2019).

#### 5.1.14 Breviscapine

The first record of Erigeron breviscapus (Vant) Hand-Mazz, commonly known as “Dengzhanhua,” can be traced back to the book “Dian Nan Ben Cao.” Breviscapine, extracted from Dengzhanhua, is one of its major active monomers (Gao et al., 2017; Wen et al., 2021b; Zhu et al., 2022b). Currently, evidence-based studies have shown that Breviscapine has significant clinical efficacy and good safety for treating stroke (Zhi et al., 2018; Lyu et al., 2020). Breviscapine has various functions, such as reducing autophagy of neurons and astrocytes in the ischemic penumbra, promoting nitric oxide synthesis in endothelial cells, reducing the synthesis of vascular wall and thromboxane A2, inhibiting platelet activation and aggregation, and reducing the risk of thrombosis (Li et al., 2020c; Chen et al., 2022b). In both *in vivo* and *in vitro* models of cerebral ischemia, the neuroprotective mechanism of Dengzhanhua may be related to reducing inflammatory responses, decreasing cell apoptosis, alleviating brain edema, promoting angiogenesis, and increasing brain-derived neurotrophic factor (Pengyue et al., 2017; Long et al., 2020; Chen et al., 2022b). A recent study indicated that Breviscapine could significantly improve the cognitive function of rats with CCI and alleviate the pathological damage of ischemic neurons. The mechanism of this effect may be related to the inhibition of NLRP3 inflammasome activation and cell pyroptosis pathways in brain tissue (Wang et al., 2020).

#### 5.1.15 Sulforaphane

Sulforaphane (SFN), mainly derived from cruciferous vegetables such as broccoli and mustard greens (Vanduchova et al., 2019), is an isothiocyanate with strong bioactivities such as anticancer, antioxidative, immunomodulatory, antibacterial, and anti-inflammatory effects (Huang et al., 2019; Yagishita et al., 2019; Wu et al., 2020c; Nandini et al., 2020; Schepici et al., 2020; Kaiser et al., 2021; Xie et al., 2021). In improving brain ischemia, SFN was found to reduce cortical neuron damage caused by oxygen-glucose deprivation (OGD)/reoxygenation after primary culture of cortical neurons from Sprague Dawley (SD) rats aged 0–1 day (Li et al., 2022c). SFN also significantly inhibited oxidative stress damage and peripheral neuronal degeneration in traumatic brain injury (TBI) models (Calabrese and Kozumbo, 2021). SFN increased the expression of Bcl-2 and decreased the expression of cleaved caspase-3 to inhibit apoptosis of neurons after OGD (Alfieri et al., 2013). Additionally, SFN produced neuroprotective effects through the activation of phosphatidylinositol 3-kinase (PI3K)/Akt signaling pathway. As an effective antioxidant, SFN can activate the Nrf2-ARE signaling pathway to improve oxidative stress and exert neuroprotective effects in epilepsy-induced brain damage (Sun et al., 2017). David Vauzour et al. found that SFN protected neurons from Parkinson’s disease by activating the Nrf2 pathway to upregulate the expression of antioxidant genes (Wei et al., 2022). Soane et al. found that SFN protected hippocampal neurons of mice from death caused by OGD or hemoglobin (Soane et al., 2010). Zhang et al. confirmed that SFN exerted neuroprotective effects by increasing the expression of Nrf2 and HO-1 (Ping et al., 2010). Deng et al. (2012) showed that SFN induced the nuclear translocation of Nrf2 and increased the expression of HO-1 to protect neurons from dopamine neurotoxicity damage via the PI3K/Akt pathway. In animal models of Parkinson’s disease, SFN was found to counteract the toxic effects of 6-hydroxydopamine-1-methyl-4-phenyl-1,2,3,6-tetrahydropyridine-mediated damage and reduce degeneration and death of striatal dopaminergic neurons (Siebert et al., 2009; Jazwa et al., 2011). Vauzour et al. (2010) found that SFN could resist the toxic effects of 5-S-cysteinyl-dopamine on cortical neurons by activating extracellular signal-regulated kinase 1/2, Nrf2, and detoxifying enzymes. In terms of inhibiting inflammasomes, the latest study found that SFN can improve neurological function deficits, reduce cerebral infarct volume, and alleviate the inflammatory response after cerebral ischemia-reperfusion, thus achieving a neuroprotective effect on rat cerebral ischemia-reperfusion injury. This may be achieved by activating the JAK2-STAT3 signaling pathway, inhibiting the activation of NLRP3 inflammasomes, and reducing the inflammatory response during ischemia-reperfusion (Chang, 2017).

#### 5.1.16 Metformin

Recently, it has been found that metformin, a traditional antidiabetic drug, originally derived from a guanidine compound found in plants and modified, has the potential to reduce brain damage caused by ischemia/reperfusion injury and protect neurological function without affecting blood glucose levels (Lee et al., 2021; Sharma et al., 2021). This may be due to its ability to activate AMPK-induced autophagy, reduce brain tissue damage, promote vascular and neural regeneration, inhibit inflammatory responses, and promote central nervous system recovery (Jia et al., 2015; Arbeláez-Quintero and Palacios, 2017). Recent studies have shown that metformin can protect against ischemia/reperfusion injury by inhibiting TLR4 and NF-κB protein expression, reducing inflammatory reactions, and providing neuroprotective effects (Liu et al., 2022). Deng et al. found that metformin preconditioning is necessary for its neuroprotective effects during the acute phase of cerebral ischemic injury. Pre-treatment with 10 mg/kg/d of metformin for 7 days significantly reduced ischemic brain damage, and the protective effect of metformin may be related to the downregulation of AMPK (Deng, 2016). In terms of inhibiting inflammasomes, Yuan et al. (2021) found that metformin has a protective effect on brain damage caused by ischemia/reperfusion, and its mechanism of action includes activating the AMPK pathway, promoting autophagy, removing damaged mitochondria through autophagy, reducing cell necrosis caused by NLRP3 inflammasome activation by damaged mitochondria, and thereby protecting against ischemia/reperfusion brain damag.

It is noteworthy that metformin, as a member of the guanidine family, was originally derived from a molecular modification of guanidine, which in turn is derived from an extract of Galega officinalis, a plant commonly known as goat’s rue. This is a classic case of using natural compounds as lead compounds for drug development. In the future, we hope to discover more natural compounds as lead compounds and develop specific inflammasome-targeting drugs, providing safe and effective new drug formulations for future clinical applications.

The mechanisms of natural plant products were summarized in Table 1.

**TABLE 1:** Summary of the mechanisms of natural plant products.

Natural plant products	Functions	Reference
Toona polyphenols	Regulate the prefrontal cortex and hippocampal MAPK pathways and NLRP3 inflammasome pathways	Zhao (2022)
Proantho Cyanidins	Downregulate the TLR4-NLRP3 inflammatory signaling pathway	Yang (2022)
Panax notoginseng saponins	Downregulate the NF-κB protein activation and NLRP3 inflammasome formation	Zhou et al. (2021)
Neferine	Inhibite the activation of NLRP3 inflammasome	Huang et al. (2021b)
Salvianolic acid	Inhibit the activation and cell apoptosis of small glial cells through the P2X7/NLRP3/GSDMD pathway	Ma (2021)
Salidroside	Inhibit the activation of NLRP3 inflammasome and TLR4/NF-κB signaling pathway	Liu (2022)
α-Asarone	Reduce the excessive activation of NLRP3 inflammasomes	Fei-Fei et al. (2022)
Acacia	Inhibite of NLRP3 inflammasome	Juan et al. (2023)
Curcumin	Inhibite the NLRP3/Caspase-1 signaling axis and NLRP3 inflammasome function	Zhang et al. (2023)
Morin	Inhibite the TXNIP/NLRP3/Caspase-1 signaling pathway	Xue et al. (2023)
Ginsenoside Rd	Decrease the expression levels of FoxO1 and Keap1, and activating the Nrf2 antioxidant signaling pathway	Yao (2022)
Paeoniflorin	Inhibite the activation of NLRP3 and Caspase-1, reduce microglial cell activation, and inhibite neuronal necrosis	Aikmu et al. (2021)
Astragaloside IV	Inhibite NF-κB protein phosphorylation and NLRP3 inflammasome activation	Tang et al., 2019c; Tang (2019)
Breviscapine	Inhibite NLRP3 inflammasome activation and cell pyroptosis pathways	Wang et al. (2020)
Sulforaphane	Activate the JAK2-STAT3 signaling pathway and inhibite the activation of NLRP3 inflammasomes	Chang (2017)
Metformin	Reduce cell necrosis caused by NLRP3 inflammasome activation	Yuan et al. (2021)

## 6 Prospects

This review provides a summary of the neuroinflammatory mechanism mediated by the NLRP3 inflammasome in the context of cerebral ischemia-reperfusion injury. Accumulating evidence from both *in vitro* and *in vivo* studies supports the involvement of the inflammasome signaling pathway in the pathogenesis of cerebral ischemia-reperfusion injury, demonstrated by direct activation of inflammasomes in animal models and patients. Importantly, the inflammasome-mediated inflammatory mechanism persists from the early to late stages of cerebral ischemia-reperfusion injury, indicating its potential as a therapeutic target. Modulating the activity of the inflammasome may help mitigate its impact on pathogenic cells, such as microglia and neurons, and the associated structural damage observed in the progression of cerebral ischemic disease. Currently, clinically approved therapeutic drugs like Anakinra, Canakinumab, and Ilorinase effectively target IL-1β. Recent clinical studies have shown that Canakinumab can improve cardiovascular conditions, including atherosclerosis, an independent risk factor for stroke. Additionally, clinical trials are underway for drugs that neutralize IL-18, such as Tadekinig alfa and GSK1070806. Nevertheless, it is crucial to consider potential side effects when targeting the NLRP3 inflammasome and to ensure patient protection against pathogenic microbial infections while reducing inflammatory activity. Finding therapeutic interventions that selectively target specific types of inflammasome complexes may offer enhanced safety and long-term efficacy, particularly for neurodegenerative diseases like cerebral ischemia-reperfusion injury.

Currently, there is growing interest in exploring natural plant products for the treatment of NLRP3 inflammasome-mediated neuroinflammation in cerebral ischemia/reperfusion injury. These natural products mainly consist of flavonoids, alkaloids, polysaccharides, quinones, terpenes, lignans, coumarins, phenolic acids, and saponins. Most studies conducted thus far have relied on *in vitro* and *in vivo* experiments. While certain natural compounds, such as total glucosides of paeony, have been utilized in cerebral ischemia treatment, there is a lack of large-scale, long-term clinical studies to validate their efficacy. This calls for further investigation. The aim of reviewing natural compounds is to identify potential lead compounds capable of inhibiting the NLRP3 inflammasome in cerebral ischemia. These findings can subsequently contribute to the development of specific inflammasome inhibitors. A comprehensive management strategy for patients with cerebral ischemia represents a promising direction for future research. The key focus for both basic researchers and clinical physicians is to devise rational treatment plans that can alleviate complications by targeting inflammatory processes. Ultimately, understanding the mechanisms underlying inflammatory processes in cerebral ischemia/reperfusion injury will open up new avenues for the comprehensive treatment of cerebral ischemia and its associated complications.

This review reveals a structural commonality among several compounds - the presence of a phenol unit. Examples of such compounds include chimonanthus polyphenols, anthocyanins, ellagic acid, curcumin, and luteolin. Based on this finding, it is speculated that the inhibition of NLRP3 inflammasome activity by polyphenolic compounds and their metabolites mainly depends on the type, number, and arrangement of functional groups on the core structure. This, in turn, affects their anti-inflammatory activity. It suggests that future design of NLRP3 inflammasome inhibitors can be based on this class of phenolic structures, necessitating further research and structural modifications by future investigators.

Furthermore, another class of compounds based on sapogenin glycosides, such as total saponins of Panax notoginseng, salidroside, ginsenoside Rd, paeoniflorin, and astragaloside IV, is identified. These compounds exhibit structural characteristics that involve spirosolane, related steroidal compounds, or triterpenes. From a structure-activity standpoint, the complex nature of these structures requires further clarification of their structure-activity relationship in inhibiting NLRP3 inflammasome activity. This could provide new perspectives for structural modifications from different angles in future studies.
